# Functional Expression of Choline Transporters in Human Neural Stem Cells and Its Link to Cell Proliferation, Cell Viability, and Neurite Outgrowth

**DOI:** 10.3390/cells10020453

**Published:** 2021-02-20

**Authors:** Yosuke Fujita, Tomoki Nagakura, Hiroyuki Uchino, Masato Inazu, Tsuyoshi Yamanaka

**Affiliations:** 1 Department of Anesthesiology, Tokyo Medical University, 6-7-1 Nishishinjuku, Shinjuku-ku, Tokyo 160-0023, Japan; yosuke618@gmail.com (Y.F.); tomoki_5029@yahoo.co.jp (T.N.); huchino@tokyo-med.ac.jp (H.U.); 2 Institute of Medical Science, Tokyo Medical University, 6-1-1 Shinjuku, Shinjuku-ku, Tokyo 160-8402, Japan; 3 Department of Molecular Preventive Medicine, Tokyo Medical University, 6-1-1 Shinjuku, Shinjuku-ku, Tokyo 160-8402, Japan; yamanaka@rtss.co.jp

**Keywords:** choline transporter, neural stem cells, self-renewal, differentiation, choline deficiency

## Abstract

Choline and choline metabolites are essential for all cellular functions. They have also been reported to be crucial for neural development. In this work, we studied the functional characteristics of the choline uptake system in human neural stem cells (hNSCs). Additionally, we investigated the effect of extracellular choline uptake inhibition on the cellular activities in hNSCs. We found that the mRNAs and proteins of choline transporter-like protein 1 (CTL1) and CTL2 were expressed at high levels. Immunostaining showed that CTL1 and CTL2 were localized in the cell membrane and partly in the mitochondria, respectively. The uptake of extracellular choline was saturable and performed by a single uptake mechanism, which was Na^+^-independent and pH-dependent. We conclude that CTL1 is responsible for extracellular choline uptake, and CTL2 may uptake choline in the mitochondria and be involved in DNA methylation via choline oxidation. Extracellular choline uptake inhibition caused intracellular choline deficiency in hNSCs, which suppressed cell proliferation, cell viability, and neurite outgrowth. Our findings contribute to the understanding of the role of choline in neural development as well as the pathogenesis of various neurological diseases caused by choline deficiency or choline uptake impairment.

## 1. Introduction

Neural stem cells (NSCs) are undifferentiated cells that have both self-renewal and multilineage potential. NSCs proliferate in response to epidermal growth factor (EGF) and fibroblast growth factor (FGF) and produce neural progenitor cells (NPCs) [1,2]. NPCs differentiate into neurons, astrocytes, and oligodendrocytes under various regulations and are responsible for a major role in neural development [3]. NSCs have been the focus of much attention in regenerative medicine, such as stem cell transplantation for nerve injury and neurodegenerative diseases.

Recent studies have shown that the transplantation of human-induced pluripotent stem cell-derived NPCs promotes the recovery of motor function in primates with spinal cord injury [4]. However, the mechanisms by which neural stem cells maintain their self-renewal and pluripotency and undergo cell differentiation have not been fully elucidated. Investigation of this mechanism will not only lead to understanding of the developmental mechanism of the brain nervous system but also provide fundamental knowledge for the realization of regenerative medicine for brain injury and neurodegenerative diseases.

Choline is a quaternary ammonium cation, a water-soluble vitamin-like substance, and is indispensable for all cellular activities. Choline has several critical roles in biological systems. First, phosphatidylcholine (PC) and sphingomyelin (SM), which are synthesized from choline, mainly constitute the outer layer of the cell membrane lipid bilayer [5]. Second, acetylcholine (ACh), a neurotransmitter, is synthesized from choline and acetyl-CoA via choline acetylcholine transferase in the cytosol of cholinergic nerves [6].

Third, betaine is formed from choline via dehydrogenase through oxidative metabolism, which supplies an important osmolyte in the kidney [7], a methyl group to homocysteine in the methionine circuit, and is involved in the synthesis of *S*-adenosylmethionine (SAM). As a methyl donor substrate, SAM is required for DNA methylation and has attracted attention as a factor implicated in the regulation of epigenetics [8]. Therefore, choline is taken up into cells and then metabolized in three pathways and is also involved in various cellular functions.

Previous studies identified three major types of choline transporter proteins [9]. High-affinity choline transporter 1 (CHT1/SLC5A7) is expressed in cholinergic nerves and is associated with ACh synthesis [10]. CHT1 transports choline from the extracellular space driven by the Na^+^ concentration gradient. Choline transporter-like proteins (CTL1–5/SLC44A1–5) are expressed in various types of organs [11]. Among them, choline transport by CTL1 is characterized by intermediate-affinity, Na^+^-independence, and pH-dependence [12].

CTL1 is ubiquitously expressed in various types of organs and tumors, including the brain, microvascular endothelium, and placenta, and is responsible for extracellular choline uptake in the cell membrane [13,14]. CTL2 is also expressed in various types of organs and tumors, and it regulates the transport of choline to the mitochondria and is involved in DNA methylation through SAM biosynthesis via choline oxidation in the inner mitochondrial membrane [15,16,17,18]. Poly-specific organic cation transporters (OCT1-3/SLC22A1-3) have a low affinity for choline, and they also transport other cationic compounds [19].

Several studies reported on the relevance of choline in neural development. First, a maternal choline supply is necessary for mammalian development, and CTL1 and CTL2, which are expressed in trophoblast cells, are responsible for the choline supply to the fetus [14]. In neural development, the demand for choline is thought to increase due to cell proliferation. In fact, choline supplementation during pregnancy stimulates hippocampal cell proliferation and promotes adult neurogenesis in the dentate gyrus of rodent models [20,21]. Conversely, choline deficiency inhibits fetal cell proliferation in the hippocampus and induces apoptosis [22,23].

Adequate choline supply not only promotes cell proliferation in neural development but also plays important roles in neural activity. For example, prenatal choline supplementation increases the size of cholinergic neuron in the basal forebrain and even enhances ACh synthesis and release from neurons [24,25,26]. As with cell proliferation, neuron size increase and neurite outgrowth require the promotion of membrane biosynthesis. Neuronal cell membranes are composed of various types of lipids, with PC being the most abundant in mammalian cell membranes [27].

The demand for choline is predicted to increase during neural differentiation and maturation. Choline supply or its deficiency during fetal life alters gene expression in the hippocampus and cerebral cortex in rats [28]. In addition, various epigenetic pathways, including DNA methylation, are involved in neural differentiation, and choline supply into NSCs may be responsible for their regulation [29]. Thus, choline and its metabolites have a crucial role in neural development; however, the molecular mechanisms of choline uptake in human neural stem cells (hNSCs) are not fully understood. Therefore, it is important to clarify the molecular and functional characteristics of choline transporters in hNSCs.

In this study, we identified transporters that mediate choline uptake in hNSCs and examined their functional analysis. We also examined the relationship between the choline uptake mechanism and cell proliferation, cell viability, and neurite outgrowth.

## 2. Materials and Methods

### 2.1. Cell Culture

hNSCs (H9 hESC-Derived, Gibco, Waltham, MA, USA, USA) were seeded on T75 flasks (Corning, New York, NY, USA) coated with defined substrate (diluted 1:100, CELLstart, Gibco, Waltham, MA, USA) and were cultured in Dulbecco’s Modified Eagle’s Medium (DMEM) F-12 (Gibco, USA) with 2% Neural Supplement (Gibco, USA), 2 mM glutamine (Gibco, USA), 20 ng/mL fibroblast growth factor-basic (bFGF, Gibco, USA), and 20 ng/mL epidermal growth factor (EGF, Gibco, USA). After about a week, the hNSCs were washed with Dulbecco’s Phosphate Buffered Saline (D-PBS) without Ca^2+^ and Mg^2+^ (WAKO, Tokyo, Japan), dissociated into single cells using dissociation reagent (Accutase, Gibco, USA), and passaged at a 1:4 ratio. All cultures were maintained in a humidified atmosphere of 5% CO_2_ and 95% air at 37 °C, and the culture medium was fully replaced every 2–3 days. hNSCs at passage number ≤7 were used in extracellular [^3^H]choline uptake experiments and at passage number ≤10 were used in all other experiments.

### 2.2. RNA Extraction and the Quantitative Real-Time Polymerase Chain Reaction (qPCR)

hNSCs were washed with D-PBS without Ca^2+^ and Mg^2+^ (WAKO), and the total RNA isolation kit (QIAshredder and RNeasy Mini kit, QIAGEN, Hilden, Germany) was used following the manufacturer’s instructions. qPCR was applied as previously described [14]. The specific primer pairs and TaqMan probes for the targets (CTL1-5, CHT1, and OCT1-3) and glyceraldehyde-3-phosphate dehydrogenase (GAPDH) as the control mRNAs were derived from human mRNA sequences (TaqMan Gene Expression Assays, Applied Biosystems, Foster City, CA, USA) (Table 1). One-step qPCR was performed on the total RNA (50 ng) with the TaqMan RNA-to-CT 1-Step Kit (Applied Biosystems). The qPCR data were analyzed with the LightCycler 96 system (Roche Diagnostics, Tokyo, Japan). The target gene expression levels were calculated relative to GAPDH by the comparative cycle-time (C_t_) method (relative mRNA expression = 2^−(Ct target–Ct GAPDH)^ × 100).

### 2.3. Western Blotting

The hNSCs were washed with D-PBS without Ca^2+^ and Mg^2+^ (WAKO, JP), and lysed in radioimmunoprecipitation assay (RIPA) buffer (Santa Cruz, Dallas, TX, USA), including 1 mM ethylenediaminetetraacetic acid (EDTA) and a protease inhibitor kept on ice. The lysates were centrifuged at 4 °C for 10 min. The supernatants were diluted in Laemmli sample buffer with 2-mercaptoethanol (Bio-Rad Laboratories, Hercules, CA, USA) and heated at 100 °C for 10 min. The samples and protein ladder marker (DynaMarker, BioDynamics Laboratory, Tokyo, Japan) were electrophoresed on a precast gel (TGX, Bio-Rad) and transferred to polyvinylidene difluoride (PVDF) membranes (Bio-Rad Laboratories).

After blocking, the membranes were incubated with antibodies using a Flex Western Device (iBind, Thermo Fisher Scientific, Waltham, MA, USA). The primary antibodies were anti-CTL1 (1 μg/mL dilution, ab110767, Abcam, Cambridge, UK) or anti-CTL2 (1 μg/mL dilution, 3D11, Abnova, Taipei, Taiwan). The secondary antibodies were anti-rabbit immunoglobulin G (IgG) (1:500 dilution, KPL074-1506, SeraCare, Milford, MA, USA) or anti-mouse IgG (1:500 dilution, KPL074-1806, SeraCare, USA). The protein bands were detected with the enhanced chemiluminescence (ECL) Prime Western Blotting Detection System (GE Healthcare Life Sciences, Marlborough, MA, USA). The luminescent images were acquired with the ChemiDoc XRS Plus System (Bio-Rad Laboratories, USA).

### 2.4. Immunocytochemistry

The hNSCs were seeded on 35 mm glass base dishes coated with poly-L-ornithine (PLO)/Laminin (IWAKI, Tokyo, Japan). Immunocytochemistry was performed as previously described [17]. The hNSCs were washed twice with D-PBS without Ca^2+^ and Mg^2+^ (WAKO, JP) and fixed with 100% methanol for 10 min at room temperature. The cells were incubated in the Flex Solution Kit (iBind, Thermo Fisher Scientific, USA) with the primary antibodies overnight at 4 °C. The primary antibodies were anti-Nestin (1:250 dilution, ab92391, Abcam, UK), anti-CTL1 (1:500 dilution, ab110767, Abcam, UK), anti-CTL2 (1:125 dilution, 3D11, Abnova, TW), anti-cytochrome c oxidase IV (COX IV) (1:500 dilution, ab16056, Abcam, UK), and anti-microtubule-associated protein 2 (MAP2) (1:300 dilution, 13-1500, Thermo Fisher Scientific, USA).

After washing with wash solution (Kirkegaard & Perry Laboratories, USA), the cells were incubated with secondary antibodies—either Alexa Fluor 488 or 568 goat anti-mouse or anti-rabbit secondary antibodies (1:500 dilution, Molecular Probes, Eugene, OR, USA), for 1 h at room temperature, and VECTASHIELD mounting medium with 4′,6-diamidino-2-phenylindole (DAPI) (Vector Laboratories, Burlingame, CA, USA) was added. Fluorescent images were obtained using an immunofluorescent microscope (FV10i-DOC, Olympus, JP or EVOS M7000, Thermo Fisher Scientific, USA).

### 2.5. Extracellular [^3^H]choline Uptake Assay

The extracellular choline uptake was analyzed using [^3^H]choline (specific activity: 2800 GBq/mmol, PerkinElmer, Waltham, MA, USA) in the same way as previously described [18]. The hNSCs were seeded at 1 × 10^4^ cells/well on 24-multiwell plates coated with PLO/Laminin (Corning, USA). The culture medium was removed and washed twice with the uptake buffer. The uptake buffer was composed of 125 mM NaCl, 1.2 mM CaCl_2_, 4.8 mM KCl, 1.2 mM KH_2_PO_4_, 5.6 mM glucose, 1.2 mM MgSO_4_, and 25 mM 4-(2-Hydroxyethyl)piperazine-1-ethanesulfonic acid (HEPES) adjusted to pH 7.4 with Tris. To adjust to the Na^+^-free condition, equimolar *N*-methyl-D-glucamine chloride (NMDG-Cl) was substituted for NaCl. 

In the saturation kinetics analysis, unlabeled choline was added to achieve the desired choline concentration, maintaining the concentration of [^3^H]choline constant at 17.4 nM. Various pH levels (pH 6.0, 6.5, 7.0, 7.5, 8.0, and 8.5) were adjusted by mixing 25 mM 2-Morpholinoethanesulfonic acid (MES) (pH 5.5) and 25 mM Tris (pH 8.5). Both buffers contained 125 mM NaCl, 1.2 mM CaCl_2_, 4.8 mM KCl, 1.2 mM KH_2_PO_4_, 5.6 mM glucose, and 1.2 mM MgSO_4_ as well. To adjust to various degrees of extracellular choline uptake inhibition, hemicholinium-3 (HC-3, Sigma-Aldrich, St. Louis, MO, USA) was used as a choline uptake inhibitor. Previous studies have shown that HC-3 inhibits CTL1 and CHT1 [12].

To terminate the choline uptake, the uptake buffer was removed and washed three times on ice. The cultures were lysed in 0.1 M NaOH with 0.1% Triton X-100. The radioactivity was measured with a liquid scintillation analyzer (Tri-Carb 2100TR, USA). The specific uptake of [^3^H]choline was calculated as the difference between the total [^3^H]choline uptake in the presence and absence of 30 mM unlabeled choline. The protein concentrations were measured using a detergent compatible (DC) Protein Assay Kit (Bio-Rad, USA).

### 2.6. Cell Proliferation and Cell Viability Assay

The hNSCs were seeded on 48- or 24-multiwell plates coated with defined substrate (diluted 1:100, CELLstart, Gibco, USA). Hemicholinium-3 (HC-3) was added 48 h after hNSCs plating, and the final culture medium in each well was 0.5 or 1.0 mL. To maintain the cell conditions, all culture media and HC-3 were changed every day. The cell numbers were measured with a cell viability assay kit (ATPLite, PerkinElmer, USA), and their luminescence was measured using a microplate reader (FilterMax F5, Molecular Devices, San Jose, CA, USA).

### 2.7. Caspase-3/7 Activity Assay

The hNSCs were seeded on 24-multiwell plates coated with the defined substrate (diluted 1:100, CELLstart, Gibco, USA). HC-3 was added 48 h after hNSC plating, and the final culture medium in each well was 1.0 mL. All culture media and HC-3 were changed every day. For measuring the Caspase-3/7 activity, the Caspase-3/7 assay kit (Caspase-Glo 3/7 Assay System, Promega, Madison, WI, USA) was used. This kit is based on the cleavage of the Z-DEVD-Aminoluciferin sequence of a luminogenic substrate by Caspase-3/7, resulting in a luminescent signal. Their luminescence was measured using a microplate reader (FilterMax F5, Molecular Devices, USA).

### 2.8. Neurite Outgrowth Assay

The hNSCs were seeded on a 35 mm glass base dish coated with PLO/Laminin (IWAKI, Japan). Cell differentiation was induced 48 h after hNSCs plating by culturing in Neurobasal Medium (Gibco, USA) with 2% B-27 Plus Supplement (Gibco, USA) and 2 mM glutamine (Gibco, USA) for 7 days. All cell differentiation media and HC-3 were changed every day. Immunofluorescence was performed with MAP2 and DAPI, as mentioned above. Images were obtained with an immunofluorescent microscope (EVOS M7000, Thermo Fisher Scientific, USA). The neurites were measured as the maximum straight length from the nucleus surface to the end in MAP2-positive cells.

### 2.9. Statistics Analysis

Statistical analysis was performed as previously described [14]. All data were presented as the mean ± standard deviation (SD). Statistical analysis software (Prism 8, GraphPad, San Diego, CA, USA) was used for the Dunnett multiple comparisons test, unpaired *t*-test analysis, and data calculations. The kinetic parameters K_m_ and Vmax were calculated by non-linear regression of the Michaelis–Menten equation and confirmed by linear regression of the Eadie–Hofstee plot. The concentration producing 50% inhibition (IC_50_) was also calculated by non-linear regression. A *p*-value of less than 0.05 was considered statistically significant. The inhibition constant (K_i_) values were calculated from the half maximal inhibitory concentration (IC_50_) values [30]: K_i_ = IC_50_/(1 + [L]/K_m_), where [L] is the concentration of radiolabeled ligand.

## 3. Results

### 3.1. Expression of Choline Transporter mRNA and Protein in hNSCs

**Figure 1:**
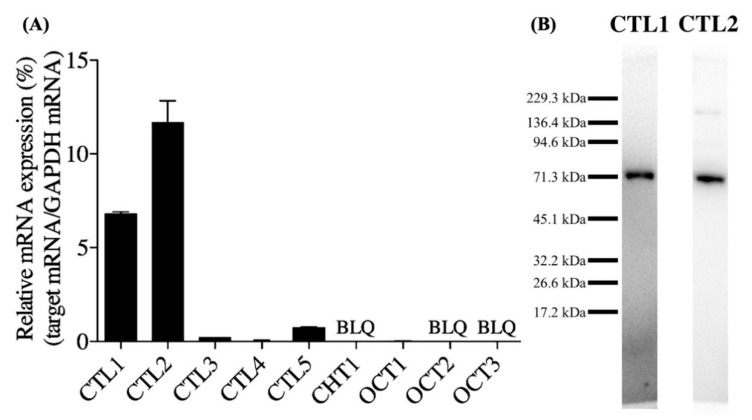
Choline transporter mRNA and protein expression in human neural stem cells (hNSCs). (**A**) Quantitative real-time polymerase chain reaction (qPCR) analysis of choline transporter-like proteins (CTL1-5), high-affinity choline transporter 1 (CHT1), and poly-specific organic cation transporters (OCT1-3). The relative mRNA expression is shown as a ratio of the target mRNA to glyceraldehyde-3-phosphate dehydrogenase (GAPDH) mRNA. Each value shows the mean ± standard deviation (SD) of three independent experiments. BLQ—below the lower limit of quantification. (**B**) Western blot analysis of CTL1 and CTL2.

**Figure 2:**
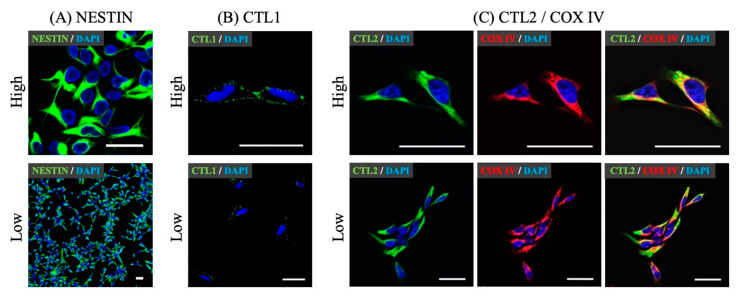
Subcellular localization of Nestin, CTL1, and CTL2 by immunocytochemistry in hNSCs. (**A**) Staining showed a neural stem cell marker, Nestin (green) and 4′,6-diamidino-2-phenylindole (DAPI) (blue). Over 95% of hNSCs expressed Nestin. (**B**) Staining showed CTL1 (green) and DAPI (blue). (**C**) Staining showed CTL2 (green), a mitochondrial marker, cytochrome c oxidase IV (COX IV) (red), and DAPI (blue). Scale bar: 20 μm. High or Low means high magnification or low magnification.

We first measured CTL1-5, CHT1, and OCT1-3 mRNA expression using qPCR (Figure 1A). CTL1 and CTL2 mRNAs were highly expressed. CTL3, CTL4, CTL5, and OCT1 mRNAs were expressed at low levels. CHT1, OCT2, and OCT3 mRNAs were not detected. CTL1 and CTL2 proteins were measured using a Western blot (Figure 1B). CTL1 and CTL2 proteins were detected as bands of approximately 70 kDa. The localization of a neural stem cell marker, Nestin, CTL1, and CTL2 proteins was investigated using immunocytochemistry. Over 95% of hNSCs expressed Nestin (Figure 2A). CTL1 was located in the cell membranes (Figure 2B). CTL2 was located in the cytoplasm, and part of its expression overlapped with a mitochondrial marker, COX IV (Figure 2C).

### 3.2. Characteristics of Extracellular [^3^H]choline Uptake in hNSCs

**Figure 3:**
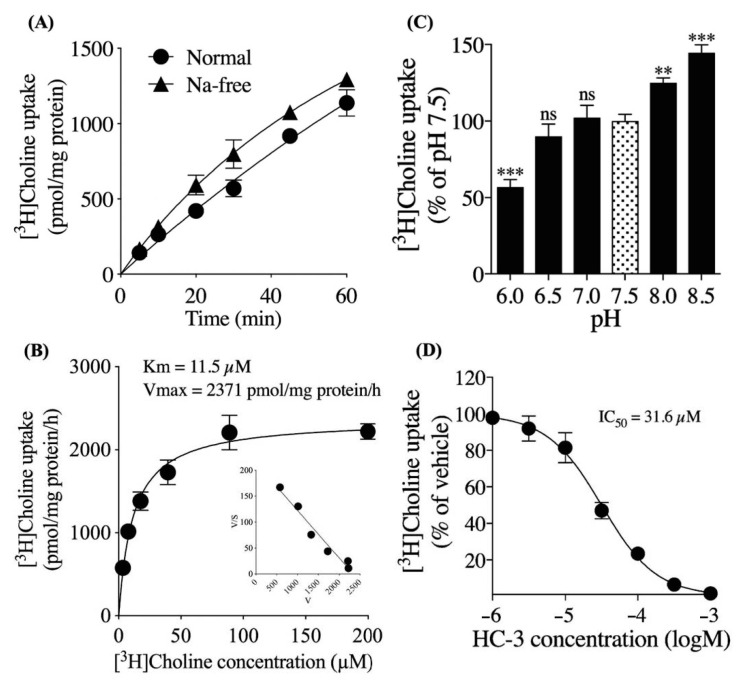
Extracellular [^3^H]choline uptake analysis in hNSCs. (**A**) Time course of 10 μM [^3^H]choline uptake in the presence and absence of extracellular Na^+^ over 60 min. (**B**) The saturation kinetics analysis of [^3^H]choline uptake. hNSCs were incubated in the uptake buffer with 3.47 to 200 μM [^3^H]choline for 20 min. Inset: Eadie–Hofstee transformations of the data. (**C**) In the effect of various degrees of extracellular pH on 10 μM [^3^H]choline uptake over 20 min. ** *p* < 0.01 and *** *p* < 0.001 denote the statistical significance vs. pH 7.5 using Dunnett multiple comparison test. ns means not significant. (**D**) [^3^H]choline uptake in various degrees of extracellular hemicholinium-3 (HC-3) concentrations. hNSCs were pre-incubated in each HC-3 concentration for 20 min. The 10 μM [^3^H]choline uptake was measured for 20 min. The data was fitted to non-linear regression analysis. Each value shows the mean ± SD of four independent experiments.

We examined the time course of 10 μM [^3^H]choline uptake in the presence or absence of extracellular Na^+^ over 60 min (Figure 3A). The amount of [^3^H]choline uptake increased linearly in a time-dependent manner. The substitution of NaCl with NMDG-Cl resulted in a slight increase in the choline uptake. The kinetics of choline uptake were investigated by non-linear regression and the Michaelis–Menten equation was fitted to the data (Figure 3B). The kinetics parameters Michaelis constant (K_m_) of 11.5 μM and maximum velocity (Vmax) of 2371 pmol/mg protein/h were calculated. 

The Eadie–Hofstee plot gave a single straight line that indicated the [^3^H]choline uptake involved a single saturable process. Next, we examined the effect of various degrees of extracellular pH on the 10 μM [^3^H]choline uptake (Figure 3C). The percentage of [^3^H]choline uptake decreased at pH 6.0 to 7.5 and increased at pH 7.5 to 8.5. We also examined the effect of HC-3, a choline uptake inhibitor, on the 10 μM [^3^H]choline uptake (Figure 3D). The [^3^H]choline uptake was inhibited in a HC-3 concentration-dependent manner with IC_50_ of 31.6 μM and calculated K_i_ of 16.9 μM.

### 3.3. Extracellular Choline Uptake Inhibition on Cellular Activities in hNSCs

**Figure 4:**
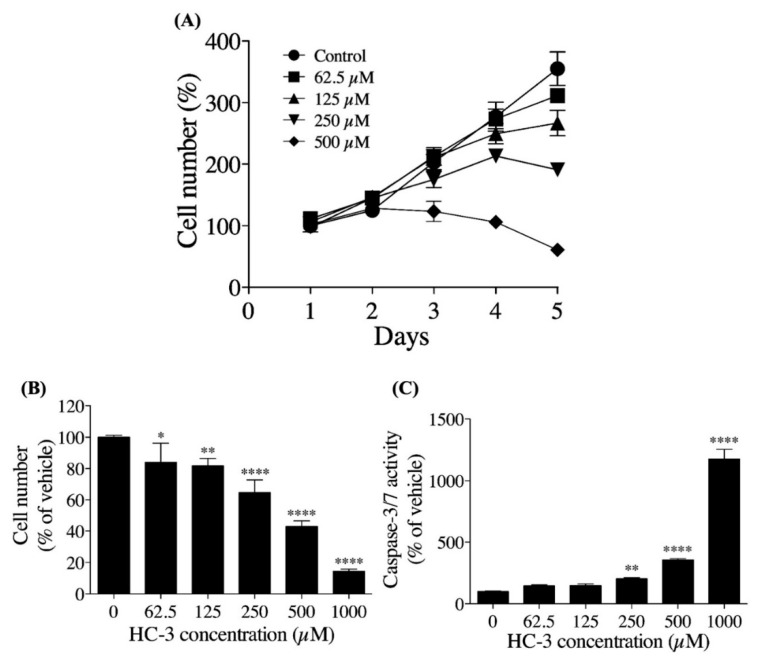
The effect of extracellular choline uptake inhibition by choline uptake inhibitor, HC-3, on cellular activities in hNSCs. (**A**) hNSCs proliferation at 5 days of cultivation in various HC-3 concentrations. hNSCs were seeded at 5 × 10^4^ cells/well on 48-multiwell plates. The results are presented as the percentage of day 1. (**B**) The hNSCs viability at 3 days of cultivation in various HC-3 concentrations. hNSCs were seeded at 5 × 10^4^ cells/well on 24-multiwell plates. The results are presented as a percentage of the 0 μM HC-3 group. (**C**) hNSCs Caspase-3/7 activity at 3 days of cultivation in various degrees of HC-3 concentrations. hNSCs were seeded at 5 × 10^4^ cells/well on 24-multiwell plates. The results are presented as the percentage of the 0 μM HC-3 group. Each value shows the mean ± SD of four independent experiments. * *p* < 0.05, ** *p* < 0.01 and **** *p* < 0.0001 denote statistical significance vs. the 0 μM HC-3 group using Dunnett multiple comparison test.

We examined the influence of extracellular choline uptake inhibition using HC-3 on cell proliferation in hNSCs (Figure 4A). Cell proliferation was suppressed in a HC-3 concentration-dependent manner. The percentage of cells began to decrease after day 5 in the 250 μM HC-3-treated group and after day 3 in the 500 μM HC-3-treated group. We also examined the influence of extracellular choline uptake inhibition on the number of viable cells and Caspase-3/7 activity over 3 days of cultivation in hNSCs (Figure 4C,D). HC-3 concentration-dependently decreased the number of viable cells and increased Caspase-3/7 activity. Caspase-3/7 activity is a hallmark of apoptosis induction [31].

**Figure 5:**
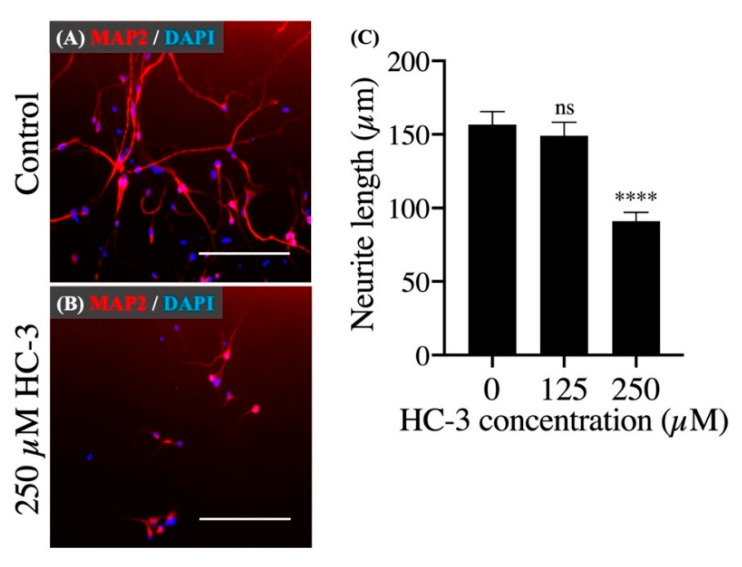
The effect of extracellular choline uptake inhibition on neurite outgrowth in hNSCs. (**A**) Cultivation in the control differentiation medium (0 μM HC-3) for 7 days. Staining shows microtubule-associated protein 2 (MAP2) (red) and DAPI (blue). Scale bar: 200 μm. (**B**) Cultivation in the differentiation medium with 250 μM HC-3 for 7 days. Staining shows MAP2 (red) and DAPI (blue). Scale bar: 200 μm. (**C**) Comparison of the neurite length in various HC-3 concentrations medium. The neurite length was measured in randomly chosen cells (30 cells) by tracing individual neurites. **** *p* < 0.0001 denotes statistical significance vs. 0 μM HC-3 group using Dunnett multiple comparison test. ns means not significant.

Finally, we investigated the influence of extracellular choline uptake inhibition on neurite outgrowth. In cell differentiation, MAP2-positive neurites appeared in both the control group and HC-3-treated group (Figure 5A,B). However, in the 250 μM HC-3-treated group, the neurite outgrowth was clearly suppressed compared to the control group (Figure 5C).

## 4. Discussion

There are several reports regarding the relationship between choline and neural development. Researchers reported that a choline-deficient diet suppressed NSC proliferation and differentiation in the mouse hippocampus [32]. Therefore, it is crucial to understand the role and dynamics of choline as well as the molecular and cellular consequences of choline deficiency in hNSCs. Thus, we first examined the types of choline transporters expressed in hNSCs. qPCR analysis showed a high expression of CTL1 and CTL2, while others demonstrated negligibly low or no expression.

CHT1, a marker for cholinergic neurons [10], was not expressed, and is thought to be expressed during neural lineage determination and differentiation maturation. The western blotting showed a major band of about 70 kDa, which is close to the predicted size of CTL1 and CTL2 [33,34]. The immunocytochemistry showed that CTL1 was clearly located in the cell membrane, while CTL2 was mainly located in the cytoplasm and partly in the mitochondria. Therefore, CTL1 may be involved in the transport of extracellular choline. Therefore, we performed a functional analysis of choline uptake in hNSCs.

The results of the extracellular choline uptake analysis showed that extracellular [^3^H]choline uptake in hNSCs was mediated by a single Na^+^-independent and intermediate-affinity uptake mechanism. CHT1 is a member of the Na^+^-dependent transporter family, which also indicates that CHT1 is not involved in the extracellular choline uptake in hNSCs [35]. The K_m_ value was 11.3 μM, which is within the range of plasma choline concentrations in healthy adults (7 to 20 μM) [36]. The extracellular [^3^H]choline uptake was dependent on the extracellular pH and increased with increasing pH. This suggests that the extracellular choline transport mechanism is H^+^ gradient-dependent. Choline uptake inhibition analysis using HC-3 showed that the K_i_ value was calculated to be 16.9 μM. Previous studies have shown that the properties of CTL1 are the K_m_ value of 10–50 μM, Na^+^-independent, and completely inhibited by HC-3 in the μM range, and our results are consistent with these properties [12,13].

Choline oxidase is found in the inner mitochondrial membrane [37]. SAM is synthesized via choline oxidase in the inner mitochondrial membrane. Previous studies have suggested that CTL2 uptakes choline in the mitochondria, synthesizes SAM, a methyl donor, via choline oxidation, and is involved in DNA methylation [18,38]. Recent studies have shown that the CTL2 protein is expressed in the mitochondria isolated from platelets, and choline uptake was confirmed [15]. CTL2 overlapped with COX IV fluorescence, a mitochondrial marker, and the CTL2 protein is likely to be expressed in the mitochondria of hNSCs. CTL2 has not yet been fully characterized, and further studies are needed. However, our main focus is the mechanism of extracellular choline uptake in hNSCs; therefore, we have not performed mitochondria-specific choline uptake analysis in this study.

Next, we investigated the influence of extracellular choline uptake inhibition on the cell proliferation, cell viability, and neurite outgrowth in hNSCs. As the degree of extracellular choline uptake inhibition increased, cell proliferation was clearly suppressed in hNSCs, and, in the 500 μM HC-3-treated group, the number of cells began to decrease early on. This result suggests that inhibition of the extracellular choline uptake resulted in an insufficient choline supply to meet the demand for PC synthesis required for cell proliferation [39]. Extracellular choline uptake inhibition increased the Caspase-3/7 activity, which is essential for the progression of apoptosis and was particularly prominent in the 1000 μM HC-3-treated group. 

A previous study reported that choline deficiency induced apoptosis via PC synthesis impairment in postmitotic primary neurons established from fetal rat brains [40]. Our results suggest that choline deficiency caused by extracellular choline uptake inhibition induced apoptosis by a similar mechanism in hNSCs. Choline, via betaine, provides methyl groups for the production of SAM, a substrate of DNA methyltransferases [8]. Studies reported that a maternal choline deficiency decreased global DNA methylation in the hippocampus of fetal mice and that global DNA methylation reduction inhibited cell proliferation in hNSCs [41,42]. Thus, intracellular choline deficiency is possibly caused by extracellular choline uptake inhibition suppressing cell proliferation in hNSCs via global DNA methylation reduction; however, this is speculative and requires further study.

Neurite outgrowth was clearly suppressed in the 250 μM HC-3-treated choline uptake inhibition group compared to the control group. Research reported that this not only increased the requirement of phosphatidylcholine but also accelerated the synthesis of phosphatidylcholine for new cell membrane synthesis during neural differentiation [43]. We suggest that extracellular choline uptake inhibition causes intracellular choline deficiency, which prevents sufficient synthesis of phosphatidylcholine for neurite outgrowth. There was no obvious suppression of neurite outgrowth in the 125 μM HC-3-treated group. Given that cell proliferation was not completely suppressed in the 125 μM HC-3-treated group, it is likely that this condition did not reach a sufficient inhibition of extracellular choline uptake to suppress neurite outgrowth. However, this study did not reveal the influence of extracellular choline uptake inhibition on the neural lineage determination in hNSCs.

In summary, CTL1 is expressed in the cell membrane and uptakes choline in hNSCs. Intracellular choline is used for the synthesis of choline metabolites, and some of them constitute the cell membrane, such as PC, or are involved in choline oxidation in the mitochondria via CTL2. In this study, we did not explore the relationship between CTL1 and ACh synthesis. However, researchers considered that CHT1, but not CTLs, provides an almost exclusive supply of choline for ACh synthesis [9]. Extracellular choline uptake inhibition by targeting CTL1 clearly suppressed the cell proliferation, cell viability, and neurite outgrowth in hNSCs. 

We considered that all of these causes were due to insufficient PC synthesis and global DNA methylation defects due to intracellular choline deficiency. However, not only the up/downregulation of self-renewal and neural differentiation but also programmed cell death, including apoptosis, are essential for the control of neural development [44]. In addition, NSCs are necessary for continuous self-renewal while maintaining multipotency. Thus, NSCs face the difficult tasks of avoiding cell cycle exit and differentiation while also avoiding tumorigenesis [45]. 

We hypothesized that the regulation of extracellular choline uptake via CTL1 is a rate-limiting step in the promotion and suppression of appropriate self-renewal in hNSCs. This suggests that the modulation of extracellular choline uptake via CTL1 is likely to be involved in neural development by regulating appropriate self-renewal, neuronal differentiation, and even cell death in hNSCs. It is possible that the amount of extracellular choline uptake is modulated by altering the CTL1 expression levels at different stages and locations in hNSCs, and further studies are required.

There are several reports of neurological disorders presumed to be caused by intracellular choline deficiency. For example, fetal spina bifida is caused by neural tube defects, which is a risk due to total choline deficiency in pregnant women [46,47]. Research also reported that impairment of neural stem/progenitor cell proliferation caused neural tube defects in mice [48]. Increased oxidative stress is a factor in the onset and progression of Alzheimer’s disease, and a study reported that oxidative stress induced by hydrogen peroxide suppressed neurite outgrowth and promoted apoptosis [49]. In contrast, the promotion of neurite outgrowth and synapse formation is considered to be essential for the recovery of neurological functions after cerebral injury [50,51]. Limiting extracellular choline uptake altered cell proliferation and neurite outgrowth in hNSCs, and our results suggest that the regulation of choline uptake via CTL1 may be partly responsible for this pathogenesis and protection. 

Although there have been no reports of diseases due to CTL1 failure, a new neurodegenerative disease caused by frameshift mutations in the SLC44A1 gene encoding CTL1 was recently described [52]. This neurodegenerative disease causes a variety of neurological symptoms, including progressive ataxia, dysarthria, dysphagia, and cognitive impairment from childhood, and brain magnetic resonance imaging (MRIs) demonstrated cerebellar atrophy and leukoencephalopathy. In their article, they reported the loss of CTL1 function and decreased choline transport in skin fibroblasts isolated from the patients. Their lipidomic analysis revealed that PC levels were maintained at the expense of other membrane phospholipids. It is not clear whether these analyses will yield similar results in hNSCs. 

However, in terms of impaired membrane homeostasis due to choline deficiency, we believe that our results partially support the possibility of CTL1 failure causing neurodegenerative disease. In the future, our findings, along with a choline-deficient hNSCs model due to extracellular choline uptake inhibition, may contribute to a better understanding of the pathogenesis of these diseases. However, our results are based on cultured hNSCs in vitro; therefore, more research is necessary to ascertain whether they are also reflected in native data in vivo. 

## Figures and Tables

**Table 1:** TaqMan gene expression assay. The gene sequence is not disclosed.

Target Gene	Accession Number	Assay ID
CTL1 (SCL44A1)	NM_080546	Hs_00223114m1
CTL2 (SCL44A2)	NM_020428	Hs_01105936m1
CTL3 (SCL44A3)	NM_001114106	Hs_00537043m1
CTL4 (SCL44A4)	NM_001178044	Hs_00228901m1
CTL5 (SCL44A5)	NM_152697	Hs_01120485m1
CHT1 (SCL5A7)	NM_021815	Hs_00222367m1
OCT1 (SLC22A1)	NM_003057	Hs_00427554m1
OCT2 (SLC22A2)	NM_003058	Hs_01010723m1
OCT3 (SLC22A3)	NM_021977	Hs_01009568m1
GAPDH	NM_002046	Hs_99999905m1

## Data Availability

No new data were created or analyzed in this study. Data sharing is not applicable to this article.
